# Association between socioeconomic level and cardiovascular risk in the Peruvian population

**DOI:** 10.11606/s1518-8787.2022056004132

**Published:** 2022-10-19

**Authors:** Stefany Katherine Cerpa-Arana, Lourdes Magaly Rimarachín-Palacios, Antonio Bernabé-Ortiz

**Affiliations:** I Universidad Científica del Sur Facultad de Ciencias de la Salud Lima Perú Universidad Científica del Sur . Facultad de Ciencias de la Salud . Lima , Lima, Perú; II Universidad Peruana Cayetano Heredia Centro de Excelencia en Enfermedades Crónicas Lima Perú Universidad Peruana Cayetano Heredia . Centro de Excelencia en Enfermedades Crónicas . Lima , Lima, Perú

**Keywords:** Chronic Disease, epidemiology, Heart Disease Risk Factors, Risk Factors, Socioeconomic Factors, Peru

## Abstract

**OBJECTIVE:**

To determine the association between socioeconomic level and the presence of obesity, hypertension and type 2 diabetes mellitus in the Peruvian population.

**METHODS:**

Secondary analysis of data from the National Demographic and Family Health Survey ( *Encuesta Nacional Demográfica y de Salud Familiar* , Endes) from 2018 to 2020. The outcomes were obesity, hypertension, and type 2 diabetes mellitus. The exposure variables were two indicators of socioeconomic status: educational level (< 7 years, 7–11 years, and 12+ years) and wealth index (in tertiles). Models were created using Poisson regression, reporting prevalence ratios (PR) and 95% confidence intervals (95%CI).

**RESULTS:**

Data from 98,846 subjects were analyzed. Mean age: 45.3 (SD: 16.0) years, and 55.5% were women. The prevalence of obesity was 26.0% (95%CI: 25.4–26.6); of hypertension, 24.9% (95%CI: 24.3–25.5); and of type 2 diabetes mellitus, 4.8% (95%CI: 4.5–5.1). In multivariate model, and compared with those with a low wealth index, those with a high wealth index had a higher prevalence of obesity (PR = 1.49; 95%CI: 1.38–1.62), hypertension (PR = 1.09; 95%CI: 1.02–1.17) and type 2 diabetes mellitus (PR = 1.72; 95%CI: 1.29–2.29). On the other hand, higher educational level was only associated with a reduction in the prevalence of obesity (PR = 0.89; 95%CI: 0.84–0.95).

**CONCLUSIONS:**

There is a differential association between the wealth index, educational level and markers of noncommunicable diseases. There is evidence of a positive association between wealth index and obesity, hypertension and type 2 diabetes mellitus, whereas educational level was only negatively associated with obesity.

## INTRODUCTION

Chronic noncommunicable diseases, including obesity, hypertension and type 2 diabetes mellitus, are on the rise, especially in low- and middle-income countries ^1^ . In Latin America, the situation is not different, and despite the existing heterogeneity, cases of obesity and type 2 diabetes mellitus have increased ^4^ .

In Peru, approximately 22.3% of the population suffers from obesity, and almost 14% have high blood pressure according to the results of the 2019 National Demographic and Health Survey ^5^ . The prevalence of type 2 diabetes mellitus reaches 7% at the national level ^6^ , with heterogeneous results between regions. Many noncommunicable diseases are determined by the interaction of genetic and metabolic factors, as well as risk factors, such as poor diet, low levels of physical activity, and aging ^7^ . All these behavioral changes have been associated with the epidemiological and nutritional transition that include social and economic growth, urbanization, globalization of technologies and food production, implying changes in the causes of morbidity and mortality in the population ^8^ .

Socioeconomic level is a total measure combining the economic and sociological part of a person’s attaintment ^9^ . This indicator can be used as a surrogate to assess the distribution of certain health risk factors, and to give an idea of the transition phase in which a given population is ongoing ^10^ . For example, the increase in the prevalence of obesity has been unequal when evaluated according to socioeconomic strata in different Latin American contexts ^11^ . On the other hand, although there are several indicators to determine socioeconomic status, two of them are the most commonly used, including educational level and wealth index ^9^ .

Thus, the present study aimed to evaluate the association at the population level between socioeconomic level, assessed using educational level and the wealth index, and the prevalence of cardiovascular risk factors (obesity, hypertension, and type 2 diabetes mellitus).

## METHODS

### Study Design

A secondary analysis was conducted using information from the National Demographic and Family Health Survey (Endes) ^12^ . The Endes is a population-based survey with national and regional representativeness, which is conducted annually by the National Institute of Statistics and Informatics ( *Instituto Nacional de Estadística e Informática* , INEI) in the 25 regions of Peru, and includes variables on poverty, fertility, violence and health. Since 2014, a specific module for noncommunicable diseases was included, and information from 2018 to 2020 was used for the present analysis.

### Selection of Participants and Sampling

The selection criteria for participants were to be aged ≥ 20 years, and able to consent to their participation in the study. Pregnant women were excluded from the present analysis.

The Endes sampling followed a two-stage random approach. In rural areas, the primary sampling units are clusters of 500 to 2,000 subjects, while the secondary sampling units are households within clusters. In urban areas, the primary sampling units are blocks or groups of blocks with more than 2,000 subjects, and an average of 140 households, and the secondary sampling units are households as in rural areas ^12^ .

### Sample Size and Power

To calculate the statistical power of this study, the OpenEpi ^13^ program was used, assuming a distribution of approximately one-third of the population in each socioeconomic level, and a difference in the prevalence of the event of interest of at least 5% (e.g., 14% obesity in the lower socioeconomic level versus 19% in the upper level) between groups to be compared. At a 95% confidence level, a power greater than 99% was obtained to find the associations of interest.

### Definition of Variables

Three were the outcomes of interest: obesity, arterial hypertension, and type 2 diabetes mellitus. Obesity was defined as a body mass index (BMI) ≥ 30 kg/m ^2^ according to international guidelines ^14^ . The presence of hypertension was characterized by a systolic blood pressure ≥ 140 mm Hg or diastolic ≥ 90 mm Hg or as a self-reported prior diagnosis according to the JNC-7. Finally, and given that the Endes does not collect blood samples for fasting blood glucose, the presence of type 2 diabetes mellitus was defined according to a self-reported previous physician diagnosis.

The exposure variable of interest was the socioeconomic level, evaluated based on two indicators: educational level and wealth index. In the case of educational level, the years of education, reported by the participant, were used and then categorized into < 7 years (compatible with completed primary school), between 7 and 11 years (compatible with completed secondary school), and ≥ 12 years (compatible with higher education). On the other hand, the wealth index is a composite measure of the standard of living in a household. It is calculated in a simple way with data collected on the respondent’s household assets and services (e.g., television, bicycle, roof, wall, floor material, etc.). This procedure is based on DHS Program techniques that are almost common to all countries participating in such program ^15^ . All these indicators were weighted, constructing a numerical wealth index that was subsequently categorized into tertiles (low, medium and high) for this analysis.

Other variables were also evaluated as potential confounders of the associations of interest. These included: sex (male *versus* female), age (in years, categorized into 20-40, 40-59, and ≤ 60 years), geographic setting (rural *versus* urban), altitude, defined based on meters above sea level (masl) of the individual’s area of residence, and further categorized (< 501, 501–2,500, and ≥ 2,501 masl), and the year in which the Endes was conducted. This last variable was introduced to account for variations in results due to the effect of the Covid-19 pandemic.

### Procedures

For the Endes’ data collection, there were several fieldwork teams, each consisting of a supervisor and fieldworkers, who were trained and standardized by the INEI staff. In recent years, data collection was done using tablets, but in 2020 it was done by telephone calls.

After the participants’ consent, data were collected using the different Endes questionnaires and instruments. Weight and height were collected with the use of scales and stadiometers calibrated by the supervisors in the study field. Blood pressure was evaluated using the OMRON automatic monitor, model HEM-713, with appropriate cuffs according to arm circumference. The blood pressure measurement was performed in duplicate, with the participant seated and the arm resting at the heart level. The first measurement was taken after a resting period of 5 minutes; and the second one, two minutes after the first measurement ^16^ .

### Statistical analysis

Stata v.16.0 (StataCorp, College Station, TX, USA) was used for statistical analysis, and the complex sampling of the study was taken into consideration for all estimates. Initially, the population characteristics were described according to socioeconomic level (educational level and wealth index), and the outcomes of interest (obesity, hypertension and type 2 diabetes mellitus). A comparison of missing values by variable and year of the Endes was also performed, because a greater loss of data in anthropometric markers was expected in 2020 due to the Covid-19 pandemic. For comparisons, the Chi-squared test was applied according to study design with the Rao-Scott second-order correction for categorical variables. In addition, the prevalence of these outcomes was estimated and reported with their respective 95% confidence intervals (95%CI).

Finally, in order to evaluate the association between socioeconomic level (educational level and wealth index) and the outcome variables, crude and adjusted models were created using Poisson regression, with robust variance, reporting the prevalence ratio (PR) and their corresponding 95% confidence intervals (95%CI). The multivariate models were adjusted for sex, age, geographical area, altitude and year of the Endes. The variance inflation factor (VIF) was used to determine the presence of collinearity due to the multiple variables included in the model; however, all values were less than 5.

### Ethics

The Endes database is publicly accessible. The INEI staff ensured the voluntary and informed participation of respondents through informed consent. The research protocol of this study was reviewed and approved by the Ethics Committee of the *Universidad Científica del Sur* complying with the Declaration of Helsinki (code: 726-2019-PRE15).

## RESULTS

### Characteristics of the Study Population

A total of 109,363 available records came from the 2018, 2019, and 2020 Endes. However, 10,517 (9.6%) records were excluded because they did not meet the inclusion criteria (9,364 because they were younger than 20 years, and 1,153 because they were pregnant women). Thus, a total of 98,846 records were analyzed, with a mean age of 45.3 years (SD: 16.0) and 52,259 (55.5%) females. Of note, a large number of missing data in the measurement of body mass index and blood pressure was found in 2020, especially due restrictions imposed by the Covid-19 pandemic.

### Population Description According to Educational Level and Well-Being Index

According to the results in Table 1 , those with high wealth index were mostly female (p < 0.001), younger than 40 years (p < 0.001), from urban area (p < 0.001) and from sites below 500 masl (p < 0.001). On the other hand, those with higher educational level were mostly female (p < 0.001), younger than 40 years (p < 0.001), from urban area (p < 0.001), and from sites below 500 masl (p < 0.001).

**Table 1:** Population description according to well-being index and educational level taking into account the study design.

	Well-being index				Educational level			
	
	Low	Medium	High	p	< 7 years	7–11 years	≥ 12 years	p
					
	(n = 31,061) n (%)	(n = 31,236) n (%)	(n = 31,948) n (%)		(n = 25,783) n (%)	(n = 36,333) n (%)	(n = 27,960) n (%)	
Sex								
Female	16,934 (54.1)	17,426 (54.6)	17,899 (56.7)	< 0.001	15,753 (61.8)	18,941 (50.9)	15,316 (53.6)	< 0.001
Male	13,719 (45.9)	13,207 (45.4)	12,911 (43.3)		9,895 (38.2)	17,058 (49.1)	12,379 (46.4)	
Age (in years)								
20–40	14,913 (40.4)	18,450 (49.0)	16,238 (41.8)	< 0.001	8,064 (22.6)	23,046 (49.8)	18,420 (55.6)	< 0.001
40–59	9,335 (33.3)	8,590 (33.3)	9,862 (34.6)		9,691 (38.3)	10,181 (35.3)	7,348 (30.7)	
≥ 60	6,547 (26.3)	3,817 (17.7)	5,101 (23.5)		8,028 (39.1)	3,106 (14.9)	2,192 (13.7)	
Geographic area								
Rural	25,631 (79.4)	5,923 (13.4)	780 (1.2)	< 0.001	15,687 (46.9)	11,326 (18.4)	3,063 (5.5)	< 0.001
Urban	5,430 (20.6)	25,313 (86.6)	31,168 (98.8)		10,096 (53.1)	25,007 (81.6)	24,897 (94.5)	
Altitude (masl)								
< 501	7,548 (26.1)	17,121 (63.0)	21,422 (79.6)	< 0.001	9,199 (46.6)	19,639 (67.4)	15,809 (71.7)	< 0.001
501–2,500	6,371 (22.2)	6,250 (15.6)	6,101 (11.1)		5,587 (19.9)	6,742 (14.1)	5,670 (12.7)	
> 2,501	17,142 (51.7)	7,865 (21.4)	4,425 (9.3)		10,997 (34.5)	9,952 (18.5)	6,481 (15.6)	
Year of the Endes								
2018	10,554 (34.2)	10,546 (34.2)	10,831 (33.5)	0.19	8,450 (33.7)	12,160 (33.0)	9,377 (33.0)	0.01
2019	10,368 (34.0)	10,461 (33.3)	10,684 (33.1)		8,275 (32.1)	11,713 (31.5)	9,339 (33.4)	
2020	10,139 (31.8)	10,229 (32.5)	10,433 (33.4)		9,058 (34.2)	12,460 (35.5)	9,244 (33.6)	

Endes: *Encuesta Nacional Demográfica y de Salud Familiar* (National Demographic and Family Health Survey); masl: meters above sea level.

### Population Description according to Cardiovascular Risk

The prevalence of obesity was 26.0% (95%CI: 25.4–26.6), and this prevalence was higher in women than in men (p < 0.001), in those between 40 and 59 years of age (p < 0.001), in urban residents (p < 0.001), in those living below 500 masl (p < 0.001), in those with 7–11 years of education (p < 0.001), and in those with higher wealth index (p < 0.001) ( Table 2 ).

**Table 2:** Population description according to cardiovascular risk (obesity, hypertension and type 2 diabetes mellitus) taking into account the study design.

	Obesity		High blood pressure		Diabetes mellitus type 2	
		
	(n = 19,939/80,943)	p	(n = 14,946/77,332)	p	(n = 3,083/90,401)	p
Sex						
Female	13,193/45,903 (29.4)	< 0.001	7,570/43,870 (22.9)	< 0.001	1,787/50,600 (4.9)	0.36
Male	6,746/35,040 (21.6)		7,376/33,462 (27.4)		1,296/39,801 (4.7)	
Age (years)						
20–40	9,719/43,473 (21.6)	< 0.001	3,155/41,582 (8.6)	< 0.001	444/48,083 (1.1)	< 0.001
40–59	7,257/23,886 (32.0)		5,416/22,761 (26.3)		1,311/27,127 (5.5)	
≥ 60	2,963/13,584 (25.5)		6,375/12,989 (54.4)		1,328/15,191 (10.9)	
Geographic area						
Rural	4,507/28,188 (15.7)	< 0.001	4,898/26,807 (21.1)	< 0.001	6,06/31,577 (2.2)	< 0.001
Urban	15,432/52,755 (29.0)		10,048/50,525 (26.1)		2,477/58,824 (5.6)	
Altitude (masl)						
< 501	11,822/39,460 (29.6)	< 0.001	7,877/37,959 (26.9)	< 0.001	1,991/43,827 (5.9)	< 0.001
501–2,500	4,039/16,137 (23.8)		2,906/15,354 (23.4)		585/18,038 (4.2)	
> 2,501	4,078/25,346 (17.5)		4,163/24,019 (20.5)		507/28,536 (2.4)	
Year of the Endes						
2018	7,460/30,895 (25.8)	0.05	5,351/29,151 (23.5)	< 0.001	936/31,036 (4.3)	0.004
2019	7,220/30,017 (25.4)		5,090/28,382 (23.1)		975/30.185 (4.8)	
2020	5,259/20,031 (27.1)		4,505/19,799 (29.5)		1,172/29.180 (5.4)	
Education level (years)						
< 7	5,029/22,477 (25.0%)	< 0.001	5,583/21,457 (31.8%)	< 0.001	1,027/25,245 (5.8%)	0.001
7–11	8,351/31,629 (28.2%)		4,813/30,313 (22.6%)		1,065/35,440 (4.4%)	
≥ 12	6,193/24,138 (25.2%)		3,595/23,093 (20.9%)		884/27,008 (4.6%)	
Well-being index						
Low	3,986/26,919 (15.0%)	< 0.001	4,662/25,561 (21.5%)	< 0.001	517/30,339 (2.2%)	< 0.001
Medium	7,429/27,032 (27.4%)		4,618/25,910 (22.6%)		1,061/30,167 (4.4%)	
High	8,524/26,992 (30.3%)		5,666/25,861 (28.0%)		1,505/29,895 (6.3%)	

On the other hand, the prevalence of hypertension was 24.9% (95%CI: 24.3–25.5), with higher frequency in males (p < 0.001), in those of older age (p < 0.001), in residents of urban areas (p < 0.001), in those living below 500 masl (p < 0.001), in those with lower educational level (p < 0.001), in those with higher wealth index (p < 0.001), and in those evaluated in the year 2020 (p < 0.001) ( Table 2 ).

Finally, the prevalence of type 2 diabetes mellitus was 4.8% (95%CI: 4.5–5.1), and was more frequent in older participants (p < 0.001), in residents of urban areas (p < 0.001), in those living below 500 masl (p < 0.001), in those with lower educational level (p < 0.001), those with a higher wealth index (p < 0.001), and in those evaluated in the year 2020 (p = 0.004) ( Table 2 ).

### Association between Socioeconomic Level, Educational Level and Cardiovascular Risk.

In multivariate model ( Table 3 ), there was an association between socioeconomic level, assessed by the wealth index, and the presence of obesity, hypertension, and type 2 diabetes mellitus. Thus, compared with participants with a low wealth index, those with a high wealth being index had a 49% higher prevalence of obesity (PR = 1.49; 95%CI: 1.38–1.62). Similarly, those with high wealth index had higher prevalence of hypertension (PR = 1.09; 95%CI: 1.02–1.17) and type 2 diabetes mellitus (PR = 1.72; 95%CI: 1.29–2.29) compared to those with low wealth index. The same finding was valid for those with the medium wealth index, except in the case of hypertension.

**Table 3:** Association between socioeconomic level and cardiovascular risk. Raw and adjusted models taking into account the study design.

	Obesity		High blood pressure		Diabetes mellitus type 2	
		
	RP _crude_ (95%CI)	RP _crude_^a^ (95%CI)	RP _crude_ (95%CI)	RP _crude_^a^ (95%CI)	RP _crude_ (95%CI)	RP _crude_^a^ (95%CI)
Education level (years)	(n = 82,845)	(n = 82,845)	(n = 79,464)	(n = 79,464)	(n = 92,294)	(n = 92,294)
< 7 (ref.)	1	1	1	1	1	1
7–11	**1.13 (1.07** – **1.19)**	1.04 (0.98–1.10)	**0.71 (0.67** – **0.75)**	0.97 (0.92–1.02)	**0.76 (0.66** – **0.88)**	0.98 (0.84–1.14)
≥ 12	1.01 (0.95–1.07)	**0.89 (0.84** – **0.95)**	**0.66 (0.62** – **0.70)**	0.94 (0.89–1.01)	**0.80 (0.69** – **0.92)**	1.06 (0.91–1.23)
Well-being index	(n = 85,544)	(n = 85,544)	(n = 81,933)	(n = 81,933)	(n = 95,002)	(n = 95,002)
Low (ref.)	1	1	1	1	1	1
Medium	**1.83 (1.73** – **1.94)**	**1.46 (1.36** – **1.58)**	1.05 (0.99–1.11)	1.04 (0.97–1.11)	**2.02 (1.69** – **2.41)**	**1.55 (1.19** – **2.01)**
High	**2.03 (1.91** – **2.15)**	**1.49 (1.38** – **1.62)**	**1.30 (1.23** – **1.37)**	**1.09 (1.02** – **1.17)**	**2.88 (2.43** – **3.42)**	**1.72 (1.29** – **2.29)**

In bold, estimates that are significant (p < 0.05).

^a^ Model adjusted for sex, age, geographic area, altitude, and year of the *Encuesta Nacional Demográfica y de Salud Familiar* (Endes).

On the other hand, in multivariate model we only found an association between educational level and obesity: those with the highest educational level were 11% less likely to present obesity (PR = 0.89; 95%CI: 0.84–0.95) compared to those with the lowest educational level ( Table 3 ). This finding was not valid for hypertension or type 2 diabetes mellitus.

## DISCUSSION

This study evidences a differential association between indicators of socioeconomic level and the presence of noncommunicable diseases: wealth index was associated with the presence of obesity, hypertension, and type 2 diabetes mellitus. Thus, those with a medium or high wealth index had a higher prevalence of any of the cardiovascular risks evaluated. However, this was not the case for educational level, as those with higher education had lower prevalence of obesity, but not association was found with the other chronic conditions studied. Finally, 1 in 4 had obesity, 1 in 5 had hypertension, and about 1 in 20 had type 2 diabetes mellitus.

A cohort study in Brazil reported that participants with high socioeconomic status had a higher risk of overweight and obesity compared to those with low socioeconomic status, and this risk was higher in those who always remained in the high socioeconomic status ^17^ . Other studies have evaluated the association between socioeconomic level and cardiovascular risk using cross-sectional studies ^18^ . This association tends to vary depending on the socioeconomic indicator used and the phase of the nutritional and epidemiological transition in which the population is. Thus, in developed countries, cardiovascular risk factors are usually found in people with low socioeconomic status ^20 , 22^ , whereas in developing countries it tends to be variable.

A previous study in Peru, using data from the CRONICAS Cohort Study, reported that the population with higher income and asset index were more likely to have obesity, whereas those with higher levels of education had a lower prevalence of obesity ^21^ . This result is consistent with our findings using nationally representative data. Another study that reviewed the association between socioeconomic status, education and obesity in Peruvian women showed that obesity was more frequent in women with higher socioeconomic status; in addition, there was a lower prevalence of obesity in those with higher educational levels ^23^ .

Our results seem to be in agreement with previous studies, in which the wealth index, used as a surrogate of socioeconomic level, has a much clearer association with cardiovascular risk factors than educational level. It expands these results to conditions such as hypertension and type 2 diabetes mellitus that have been reported in international studies ^24^ . The apparently contradictory results, using the wealth index and educational level, could be supported by the nutritional transition that the Peruvian population is undergoing. Changes in dietary and physical activity patterns may be initially driven by the economic development of the country, evident in the last 30 years in Peru. Thus, the increase in the prevalence of obesity, hypertension and type 2 diabetes mellitus is due to the availability of processed and high-energy foods, associated with a sedentary lifestyle that is initially seen in the groups with higher economic income, and then affects those with lower income, with a subsequent reduction in the prevalence of these cardiovascular risk factors in the better educated groups ^28^ . This seems to be clear for obesity, but is not yet visible for hypertension or type 2 diabetes mellitus.

Our study could help to define prevention strategies aimed at the population most vulnerable to chronic diseases, usually the most economically vulnerable; however, there is still a higher prevalence of these risk factors in those with a medium-high socioeconomic level. Current evidence suggests that the impact of the transition is being observed more in rural areas than in urban areas, especially in the case of obesity. ^29^ Previous results suggest that Peru is in a post-transition epidemiological stage, in which more than 80% of deaths are attributed to non-communicable diseases ^30^ , which may pose a greater challenge to the health system. The interventions generated should be context-specific, especially where people may not have an appropriate access to the health system (rural and marginal urban areas), which is where the problem of noncommunicable conditions will be at the end of the transition.

This paper utilized data from a population-based study with regional and national representativeness to assess the associations of interest. However, there are some limitations that should be highlighted. First, as a cross-sectional study, it only allowed us to determine association and not causality between exposures and outcomes. Although temporality could be an issue, the probability of reverse causality in this analysis is almost negligible. Second, diabetes mellitus was assessed by self-report and not objectively. It is known that only 50% of the population with diabetes are aware of their condition, and getting tested probably depends on the socioeconomic status of the individuals, so this could have an effect on the reported associations. However, our findings show similar results to other studies. Finally, other variables of interest, potential confounders such as diet or physical activity levels, were not included because they are not routinely collected by the Endes.

In conclusion, there is a differential association between wealth index, educational level and markers of noncommunicable diseases. There is evidence of positive association between wealth index and obesity, hypertension and type 2 diabetes mellitus, while educational level was only negatively associated with obesity.
