# Supplementary material for: Association between socioeconomic level and cardiovascular risk in the Peruvian population
Source: Rev Saude Publica. 2022 Oct 19;56:91. doi: 10.11606/s1518-8787.2022056004132 (PMC9586518; doi:10.11606/s1518-8787.2022056004132)
Supplement: Translation

# Translation: Asociación entre nivel socioeconómico y riesgo cardiovascular en la población peruana

**Authors:** Stefany Katherine Cerpa-Arana, Lourdes Magaly Rimarachín-Palacios, Antonio Bernabé-Ortiz

**Affiliations:** I Perú Universidad Científica del Sur. Facultad de Ciencias de la Salud. Lima, Lima, Perú; II Perú Universidad Peruana Cayetano Heredia. Centro de Excelencia en Enfermedades Crónicas. Lima, Lima, Perú

**Keywords:** Enfermedad Crónica, epidemiología, Factores de Riesgo de Enfermedad Cardiaca, Factores de Riesgo, Factores Socioeconómicos, Perú

## Abstract

**OBJETIVO:**

Determinar la asociación entre el nivel socioeconómico y la presencia de obesidad, hipertensión y diabetes mellitus tipo 2 en la población peruana.

**MÉTODOS:**

Análisis de datos secundarios de la Encuesta Nacional Demográfica y de Salud Familiar (Endes) del 2018 al 2020. Las variables dependientes fueron obesidad, hipertensión y diabetes mellitus tipo 2, y las variables independientes fueron dos indicadores de nivel socioeconómico: el nivel educativo (< 7 años, 7–11 años y 12+ años) y el índice de bienestar (en terciles). Se crearon modelos usando regresión de Poisson, reportando razones de prevalencia (RP) e intervalos de confianza al 95% (IC95%).

**RESULTADOS:**

Se analizaron los datos de 98.846 sujetos, edad promedio: 45,3 (DE: 16,0) años, y 55,5% fueron mujeres. La prevalencia de obesidad fue del 26% (IC95% 25,4–26,6); la de hipertensión, 24,9% (IC95% 24,3–25,5); y la de diabetes mellitus tipo 2, 4,8% (IC95% 4,5–5,1). En modelo multivariable y comparado con los de índice de bienestar bajo, aquellos con índice de bienestar alto tenían mayor prevalencia de obesidad (RP = 1,49; IC95% 1,38–1,62), de hipertensión (RP = 1,09; IC95% 1,02–1,17) y de diabetes mellitus tipo 2 (RP = 1,72; IC95% 1,29–2,29). De otro lado, mayor nivel educativo sólo se asoció a una reducción en la prevalencia de obesidad (RP = 0,89; IC95% 0,84–0,95).

**CONCLUSIONES:**

Existe asociación diferencial entre el índice de bienestar, nivel educativo y marcadores de enfermedades no transmisibles: hay evidencia de asociación positiva entre el índice de bienestar y obesidad, hipertensión y diabetes mellitus tipo 2, mientras que el nivel educativo solo estuvo asociado de forma negativa a obesidad.

## INTRODUCCIÓN

Las enfermedades crónicas no transmisibles, entre las que se encuentran la obesidad, hipertensión y diabetes mellitus tipo 2, están en aumento, especialmente en países de ingresos bajos y medios^1^ . En Latinoamérica, la situación no es diferente, y a pesar de la heterogeneidad existente, se han incrementado los casos de obesidad y diabetes mellitus tipo 2^4^ .

En Perú se estima que el 22,3% de la población sufre de obesidad y que casi el 14% presenta hipertensión arterial según los resultados de la Encuesta Nacional Demográfica y de Salud del 2019^5^ , mientras que la prevalencia de diabetes mellitus tipo 2 llega al 7% a nivel nacional^6^ , todas con resultados variables entre regiones. Muchas de las enfermedades no transmisibles se ven determinadas por la interacción de factores genéticos y metabólicos, así como de factores de riesgo, como la mala alimentación, los bajos niveles de actividad física y el envejecimiento^7^ . Todos estos cambios conductuales han sido asociados a la transición epidemiológica y nutricional que incluyen el crecimiento social y económico, la urbanización, la globalización de las tecnologías y la producción de alimentos, implicando cambios en las causas de morbilidad y mortalidad en la población^8^ .

El nivel socioeconómico es una medida total que combina la parte económica y sociológica de la preparación laboral de una persona^9^ . Este indicador puede ser usado como un surrogado para evaluar la distribución de ciertos factores de riesgo para la salud y dar una idea de la fase de transición en la que determinada población se encuentra^10^ . Por ejemplo, el incremento en la prevalencia de obesidad ha sido desigual cuando se evalúa según estratos socioeconómicos en diferentes contextos latinoamericanos^11^ . De otro lado, aunque existen varios indicadores para determinar el nivel socioeconómico, dos de ellos son los más comúnmente usados, incluyendo nivel educativo e índice de bienestar^9^ .

De esta manera, el objetivo del presente estudio fue evaluar la asociación a nivel poblacional entre el nivel socioeconómico, evaluado usando el nivel educativo y el índice de bienestar, y la prevalencia de factores de riesgo cardiovascular (obesidad, hipertensión y diabetes mellitus tipo 2).

## MÉTODOS

### Diseño de Estudio

Se realizó un análisis secundario usando la información de la Encuesta Nacional Demográfica y de Salud Familiar (Endes)^12^ . La Endes es un estudio de base poblacional con representatividad nacional y regional, que es realizado anualmente por el Instituto Nacional de Estadística e Informática (INEI) en las 25 regiones del Perú, y que incluye variables de pobreza, fertilidad, violencia y salud. Desde el año 2014 se incluyó un módulo específico para enfermedades no transmisibles, y para el presente análisis se utilizó la información del 2018 al 2020.

### Selección de Participantes y Muestreo

Los criterios de selección de los participantes fueron tener edad ≥ 20 años y ser capaces de dar consentimiento para su participación en el estudio. Las mujeres gestantes fueron excluidas del presente análisis.

El muestreo de la Endes sigue un diseño aleatorio bietápico. En las áreas rurales, las unidades primarias de muestreo son los conglomerados de entre 500 y 2.000 sujetos, mientras que las unidades secundarias de muestreo son las viviendas dentro de los conglomerados. En las áreas urbanas, las unidades primarias de muestreo son las cuadras o grupos de cuadras con más de 2.000 sujetos y un promedio de 140 viviendas, y las unidades secundarias de muestreo son las viviendas como en las áreas rurales^12^ .

### Tamaño de Muestra y Potencia

Para calcular la potencia estadística de este estudio se utilizó el programa OpenEpi^13^ , asumiendo una distribución de aproximadamente un tercio de la población en cada nivel socioeconómico, y una diferencia en la prevalencia del evento de interés de al menos 5% (p. ej. 14% de obesidad en el nivel socioeconómico inferior *versus* 19% en el nivel superior) entre grupos a comparar. Con un nivel de confianza del 95%, se obtuvo una potencia mayor al 99% para encontrar las asociaciones de interés.

### Definición de Variables

Tres fueron las variables dependientes de interés: obesidad, hipertensión arterial y diabetes mellitus 2. La obesidad se definió como un índice de masa corporal (IMC) ≥ 30 kg/m^2^según las guías internacionales^14^ . La presencia de hipertensión se caracterizó por una presión arterial sistólica ≥ 140 mm Hg o diastólica ≥ 90 mm Hg o como autorreporte de diagnóstico previo de acuerdo al JNC-7. Finalmente, y dado que la Endes no colecta muestras de sangre para glucemia en ayunas, la presencia de diabetes mellitus tipo 2 se definió de acuerdo al autorreporte de diagnóstico previo, hecho por un médico.

La variable de exposición de interés fue el nivel socioeconómico, evaluado a partir de dos indicadores: nivel educativo e índice de bienestar. En el caso del nivel educativo se usó los años de educación reportado por el participante y luego categorizado en < 7 años (compatible con primaria completa), entre 7 y 11 años (compatible con secundaria completa), y ≥ 12 años (compatible con educación superior). De otro lado, el índice de bienestar es una medida compuesta del estándar de vida en una vivienda. Se calcula en forma sencilla con datos colectados sobre las posesiones y servicios del hogar del encuestado (p. ej. televisión, bicicleta, material del techo, de las paredes, del piso, etc.). Este procedimiento se realiza con base en las técnicas del Programa DHS que es casi común a todos los países que participan en dicho programa^15^ . Todos estos indicadores fueron ponderados, construyéndose un índice de bienestar numérico que fue posteriormente categorizado en terciles (bajo, medio y alto) para este análisis.

Otras variables fueron también evaluadas como potenciales confusores de las asociaciones planteadas. Estas incluyeron: sexo (varón *versus* mujer), edad (en años, categorizada en 20–40, 40–59, y ≤ 60 años), ámbito geográfico (rural *versus* urbano), altitud, definido con base en metros sobre el nivel del mar (msnm) de la zona de residencia del individuo y posteriormente categorizado (< 501, 501–2.500, y ≥ 2.501 msnm), y el año en que se realizó la Endes. Esta última variable fue introducida para tomar en cuenta variaciones de resultados debido al efecto de la pandemia del covid-19 en la realización de la encuesta.

### Procedimientos

Para la fase de recolección de información en la Endes se contó con varios equipos de trabajo de campo, cada uno conformado por un supervisor y encuestadores, que fueron capacitados y estandarizados por el personal del INEI. En los últimos años, la colección de los datos se hizo por medio de tabletas, pero en el 2020 se realizó por llamadas telefónicas.

Tras el consentimiento de los participantes, se procedió a recolectar los datos usando los diferentes cuestionarios e instrumentos de la Endes. El peso y la talla fueron colectados con el uso de balanzas y tallímetros calibrados por los supervisores en el terreno de estudio. La presión arterial se evaluó usando el tensiómetro automático OMRON, modelo HEM-713, con manguitos apropiados de acuerdo a la circunferencia del brazo. La medida de presión arterial fue realizada en duplicado, con el participante sentado y el brazo en reposo a la altura del corazón. La primera medida fue tomada después de 5 minutos de reposo; y la segunda, dos minutos después de la primera^16^ .

### Análisis Estadístico

Para el análisis estadístico se utilizó el programa Stata v.16.0 (StataCorp, College Station, TX, USA) y se tomó en consideración el muestreo complejo del estudio para todas las estimaciones. Inicialmente, se describió a la población según el nivel socioeconómico (nivel educativo e índice de bienestar) y las variables dependientes de interés (obesidad, hipertensión y diabetes mellitus tipo 2). Se realizó además una comparación de los valores faltantes por variable y año de la Endes, debido a que se esperaba una mayor pérdida de datos en los marcadores antropométricos en el 2020 debido a la pandemia del covid-19. Para las comparaciones se aplicó la prueba de chi-cuadrado según diseño del estudio con la corrección de segundo orden de Rao-Scott para variables categóricas. Además, se estimó la prevalencia de dichas variables dependientes reportando los intervalos de confianza al 95% (IC95%).

Por último, para evaluar la asociación entre nivel socioeconómico (nivel educativo e índice de bienestar) y las variables resultado se crearon modelos crudos y ajustados utilizando la regresión de Poisson, con varianza robusta, reportándose la razón de prevalencia (RP) y sus IC95% correspondientes. Los modelos multivariables fueron ajustados por sexo, edad, ámbito geográfico, altitud y año de la Endes. Se utilizó el valor de inflación de la varianza (VIF) para determinar la presencia de colinealidad debido a las múltiples variables incluidas en el modelo, sin embargo, todos los valores fueron inferiores a 5.

### Ética

La base de datos de la Endes es de acceso público, dicho estudio aseguró la participación voluntaria e informada de los encuestados mediante el consentimiento informado. El protocolo de investigación de este estudio fue revisado y aprobado por el Comité de Ética de la Universidad Científica del Sur cumpliendo con la Declaración de Helsinki (código: 726-2019-PRE15).

## RESULTADOS

### Características de la Población de Estudio

Un total de 109.363 registros disponibles proceden de las Endes del 2018, 2019 y 2020. Sin embargo, se excluyeron 10.517 (9,6%) registros por no cumplir los criterios de inclusión (9.364 por ser menores de 20 años, y 1.153 por ser mujeres gestantes). Así, se analizaron un total de 98.846 registros, con una media de edad de 45.3 años (DE: 16,0) y 52.259 (55,5%) mujeres. De importancia, hubo un gran número de datos faltantes en la medición del índice de masa corporal y de la presión arterial en el 2020, debido especialmente a las restricciones de la pandemia por el covid-19.

### Descripción de la Población Según el Nivel Educativo e Índice de Bienestar

De acuerdo con los resultados de la Tabla 1 , aquellos con alto índice de bienestar fueron mayormente mujeres (p < 0,001), menores de 40 años (p < 0,001), del área urbana (p < 0,001) y de sitios por debajo de los 500 msnm (p < 0,001). De otro lado, aquellos con mayor nivel educativo fueron mayormente mujeres (p < 0,001), menores de 40 años (p < 0,001), del ámbito urbano (p < 0,001), y de sitios por debajo de los 500 msnm (p < 0,001).

**Tabla 1:** Descripción de la población según índice de bienestar y nivel educativo tomando en cuenta el diseño del estudio.

	Índice de bienestar				Nivel educativo			
	
	Bajo	Medio	Alto	p	< 7 años	7–11 años	≥ 12 años	p
					
	(n = 31.061) n (%)	(n = 31.236) n (%)	(n = 31.948) n (%)		(n = 25.783) n (%)	(n = 36.333) n (%)	(n = 27.960) n (%)	
Sexo								
Mujer	16.934 (54,1)	17.426 (54.6)	17.899 (56,7)	< 0.001	15.753 (61,8)	18.941 (50,9)	15.316 (53,6)	< 0,001
Varón	13.719 (45,9)	13.207 (45,4)	12.911 (43,3)		9.895 (38,2)	17.058 (49,1)	12.379 (46,4)	
Edad (años)								
20–40	14.913 (40,4)	18.450 (49,0)	16.238 (41,8)	< 0,001	8.064 (22,6)	23.046 (49,8)	18.420 (55,6)	< 0,001
40–59	9.335 (33,3)	8.590 (33,3)	9.862 (34,6)		9.691 (38,3)	10.181 (35,3)	7.348 (30,7)	
≥ 60	6.547 (26,3)	3.817 (17,7)	5.101 (23,5)		8.028 (39,1)	3.106 (14,9)	2.192 (13,7)	
Ámbito geográfico								
Rural	25.631 (79,4)	5.923 (13,4)	780 (1,2)	< 0,001	15.687 (46,9)	11.326 (18,4)	3.063 (5,5)	< 0,001
Urbano	5.430 (20,6)	25.313 (86,6)	31.168 (98,8)		10.096 (53,1)	25.007 (81,6)	24.897 (94,5)	
Altitud (msnm)								
< 501	7.548 (26,1)	17.121 (63,0)	21.422 (79,6)	< 0,001	9.199 (46,6)	19.639 (67,4)	15.809 (71,7)	< 0,001
501–2.500	6.371 (22,2)	6.250 (15,6)	6.101 (11,1)		5.587 (19,9)	6.742 (14,1)	5.670 (12,7)	
> 2.501	17.142 (51,7)	7.865 (21,4)	4.425 (9,3)		10.997 (34,5)	9.952 (18,5)	6.481 (15,6)	
Año de la Endes								
2018	10.554 (34,2)	10.546 (34,2)	10.831 (33,5)	0,19	8.450 (33,7)	12.160 (33,0)	9.377 (33,0)	0,01
2019	10.368 (34,0)	10.461 (33,3)	10.684 (33,1)		8.275 (32,1)	11.713 (31,5)	9.339 (33,4)	
2020	10.139 (31,8)	10.229 (32,5)	10.433 (33,4)		9.058 (34,2)	12.460 (35,5)	9.244 (33,6)	

Endes: Encuesta Nacional Demográfica y de Salud Familiar; msnm: metros sobre el nivel del mar.

### Descripción de la Población de Acuerdo a Riesgo Cardiovascular

La prevalencia de obesidad fue del 26% (IC95% 25,4–26,6), y esta prevalencia fue mayor en mujeres que en varones (p < 0,001), en aquellos entre 40 y 59 años (p < 0,001), en los residentes urbanos (p < 0,001), en aquellos que vivían por debajo de los 500 msnm (p < 0,001), en aquellos con 7–11 años de educación (p < 0,001), y en aquellos con mayor índice de bienestar (p < 0,001) ( Tabla 2 ).

**Tabla 2:** Descripción de la población según riesgo cardiovascular (obesidad, hipertensión y diabetes mellitus tipo 2) tomando en cuenta el diseño del estudio.

	Obesidad		Hipertensión arterial		Diabetes mellitus tipo 2	
		
	(n = 19.939/80.943)	p	(n = 14.946/77.332)	p	(n = 3.083/90.401)	p
Sexo						
Mujer	13.193/45.903 (29,4)	< 0,001	7.570/43.870 (22,9)	< 0,001	1.787/50.600 (4,9)	0,36
Varón	6.746/35.040 (21,6)		7.376/33.462 (27,4)		1.296/39.801 (4,7)	
Edad (años)						
20–40	9.719/43.473 (21,6)	< 0,001	3.155/41.582 (8,6)	< 0,001	444/48.083 (1,1)	< 0,001
40–59	7.257/23.886 (32,0)		5.416/22.761 (26,3)		1.311/27.127 (5,5)	
≥ 60	2.963/13.584 (25,5)		6.375/12.989 (54,4)		1.328/15.191 (10,9)	
Ámbito geográfico						
Rural	4.507/28.188 (15,7)	< 0,001	4.898/26.807 (21,1)	< 0,001	6.06/31.577 (2,2)	< 0,001
Urbano	15.432/52.755 (29,0)		10.048/50.525 (26,1)		2.477/58.824 (5,6)	
Altitud (msnm)						
< 501	11.822/39.460 (29,6)	< 0,001	7.877/37.959 (26,9)	< 0,001	1.991/43.827 (5,9)	< 0,001
501–2500	4.039/16.137 (23,8)		2.906/15.354 (23,4)		585/18.038 (4,2)	
> 2501	4.078/25.346 (17,5)		4.163/24.019 (20,5)		507/28.536 (2,4)	
Año de la Endes						
2018	7.460/30.895 (25,8)	0,05	5.351/29.151 (23,5)	< 0,001	936/31.036 (4,3)	0,004
2019	7.220/30.017 (25,4)		5.090/28.382 (23,1)		975/30.185 (4,8)	
2020	5.259/20.031 (27,1)		4.505/19.799 (29,5)		1.172/29.180 (5,4)	
Nivel educativo (años)						
< 7	5029/22477 (25,0%)	< 0,001	5583/21457 (31,8%)	< 0,001	1027/25245 (5,8%)	0,001
7–11	8351/31629 (28,2%)		4813/30313 (22,6%)		1065/35440 (4,4%)	
≥ 12	6193/24138 (25,2%)		3595/23093 (20,9%)		884/27008 (4,6%)	
Índice de bienestar						
Bajo	3986/26919 (15,0%)	< 0,001	4662/25561 (21,5%)	< 0,001	517/30339 (2,2%)	< 0,001
Medio	7429/27032 (27,4%)		4618/25910 (22,6%)		1061/30167 (4,4%)	
Alto	8524/26992 (30,3%)		5666/25861 (28,0%)		1505/29895 (6,3%)	

De otro lado, la prevalencia de hipertensión arterial fue del 24,9% (IC95% 24,3–25,5), con más frecuencia en los varones (p < 0,001), en aquellos de mayor edad (p < 0,001), en los residentes de áreas urbanas (p < 0,001), en aquellos que vivían por debajo de los 500 msnm (p < 0,001), en aquellos con menor nivel educativo (p < 0,001), en aquellos con mayor índice de bienestar (p < 0,001), y en los evaluados en el año 2020 (p < 0,001) ( Tabla 2 ).

Finalmente, la prevalencia de diabetes mellitus tipo 2 fue del 4,8% (IC95% 4,5–5,1), y fue más frecuente en los participantes de mayor edad (p < 0,001), en los residentes de áreas urbanas (p < 0,001), en aquellos que vivían por debajo de los 500 msnm (p < 0,001), en aquellos con menor nivel educativo (p < 0,001), los que tenían un mayor índice de bienestar (p < 0,001), y en los evaluados en el año 2020 (p = 0,004) ( Tabla 2 ).

### Asociación entre Nivel Socioeconómico, Nivel Educativo y Riesgo Cardiovascular

En modelo multivariable ( Tabla 3 ), hubo asociación entre el nivel socioeconómico, medido mediante el índice de bienestar y la presencia de obesidad, hipertensión y diabetes mellitus tipo 2. Así, comparado con los participantes con índice de bienestar bajo, aquellos con un índice de bienestar alto tenían 49% más prevalencia de obesidad (RP = 1,49; IC95% 1,38–1,62). De la misma forma, los de índice de bienestar alto tenían mayor prevalencia de hipertensión (RP = 1,09; IC95% 1,02–1,17) y de diabetes mellitus tipo 2 (RP = 1,72; IC95% 1,29–2,29) en comparación con los de índice de bienestar bajo. El mismo hallazgo de asociación fue válido para aquellos con el índice de bienestar medio, excepto para el caso de hipertensión.

**Tabla 3:** Asociación entre nivel socioeconómico y riesgo cardiovascular. Modelos crudos y ajustados tomando en cuenta el diseño del estudio.

	Obesidad		Hipertensión arterial		Diabetes mellitus tipo 2	
		
	RP_crudo_(IC95%)	RP_ajustada_^a^(IC95%)	RP_crudo_(IC95%)	RP_ajustada_^a^(IC95%)	RP_crudo_(IC95%)	RP_ajustada_^a^(IC95%)
Nivel educativo (años)	(n = 82.845)	(n = 82.845)	(n = 79.464)	(n = 79.464)	(n = 92.294)	(n = 92.294)
< 7 (ref.)	1	1	1	1	1	1
7–11	**1,13 (1,07–1,19)**	1,04 (0,98–1,10)	**0,71 (0,67–0,75)**	0,97 (0,92–1,02)	**0,76 (0,66–0,88)**	0,98 (0,84–1,14)
≥ 12	1,01 (0,95–1,07)	**0,89 (0,84–0,95)**	**0,66 (0,62–0.70)**	0,94 (0,89–1,01)	**0,80 (0,69–0,92)**	1,06 (0,91–1,23)
Índice de bienestar	(n = 85.544)	(n = 85.544)	(n = 81.933)	(n = 81.933)	(n = 95.002)	(n = 95.002)
Bajo (ref.)	1	1	1	1	1	1
Medio	**1,83 (1,73–1,94)**	**1,46 (1,36–1,58)**	1,05 (0,99–1,11)	1,04 (0,97–1,11)	**2,02 (1,69–2,41)**	**1,55 (1,19–2,01)**
Alto	**2,03 (1,91–2,15)**	**1,49 (1,38–1,62)**	**1,30 (1,23 - 1,37)**	**1,09 (1,02 - 1,17)**	**2,88 (2,43–3,42)**	**1,72 (1,29–2,29)**

En negrita, estimados que son significativos (p < 0,05).

^a^Modelo ajustado por sexo, edad, ámbito geográfico, altitud, y año de la Encuesta Nacional Demográfica y de Salud Familiar (Endes).

De otro lado, en modelo multivariable solo se encontró asociación entre el nivel educativo y obesidad: aquellos con el mayor nivel educativo tenían el 11% menor probabilidad de presentar obesidad (RP = 0,89; IC95% 0,84–0,95) en comparación con los de menor nivel educativo ( Tabla 3 ). Este hallazgo no fue válido para hipertensión o diabetes mellitus tipo 2.

## DISCUSIÓN

Este estudio evidencia una asociación diferencial entre los indicadores de nivel socioeconómico y la presencia de enfermedades no transmisibles: el índice de bienestar estuvo asociado a la presencia de obesidad, hipertensión y diabetes mellitus tipo 2. Así, aquellos con un índice de bienestar medio o alto tuvieron mayor prevalencia de cualquiera de los riesgos cardiovasculares evaluados. Sin embargo, este no fue el caso para el nivel educativo, ya que aquellos con mayor educación tuvieron menor prevalencia de obesidad, pero no las otras condiciones crónicas estudiadas. Finalmente, 1 de cada 4 presentaron obesidad, 1 de cada 5 presentaron hipertensión, y alrededor de 1 de cada 20 tuvieron diabetes mellitus tipo 2.

Un estudio de cohorte en Brasil reportó que los participantes con nivel socioeconómico alto tuvieron mayor riesgo de sobrepeso y obesidad comparados con los de nivel bajo, y ese riego fue mayor en aquellos que siempre se mantuvieron en el nivel alto^17^ . Otros estudios han evaluado la asociación entre nivel socioeconómico y riesgo cardiovascular usando estudios transversales^18^ . Dicha asociación suele ser variable dependiendo del indicador socioeconómico usado y la fase de la transición nutricional y epidemiológica en el que la población se encuentra. Así, en los países desarrollados, usualmente los factores de riesgo cardiovascular se encuentran en las personas con bajo nivel socioeconómico^20 , 22^ , mientras que en los países en desarrollo suele ser variable.

Un estudio previo en Perú, usando la cohorte CRONICAS, reportó que la población de mayores ingreso e índice de activos tuvo más chances de tener obesidad, mientras que aquellos con niveles más altos de educación tuvieron una menor prevalencia de obesidad^21^ . Este resultado coincide con nuestros hallazgos usando datos representativos del país. Otro estudio que analizó la asociación entre nivel socioeconómico, educación y obesidad en mujeres peruanas mostró que la obesidad fue más frecuente en mujeres con posición socioeconómica más alta; además, hubo una menor prevalencia de obesidad en aquellas con mayor nivel educativo^23^ .

Nuestros resultados parecen estar acordes a estudios previos, en que el índice de bienestar, usado como un surrogado del nivel socioeconómico, tiene una más clara asociación con los factores de riesgo cardiovascular que el nivel educativo; y expande estos resultados a condiciones como la hipertensión arterial y diabetes mellitus tipo 2 que han sido reportadas en trabajos internacionales^24^ . Los resultados, aparentemente contradictorios y usando el índice de bienestar y el nivel educativo, podrían tener sustento en la transición nutricional que sufre la población peruana. Los cambios en patrones de dieta y de actividad física pueden verse empujados inicialmente por el desarrollo económico del país, el cual ha sido patente en los últimos treinta años en Perú. Así, el aumento en la prevalencia de obesidad, hipertensión y diabetes mellitus tipo 2 se debería a la disponibilidad de alimentos procesados y de alto contenido energético, asociado a un estilo de vida sedentario que se ve inicialmente en los grupos con mayor ingreso económico, para luego afectar a los de menores ingresos con la reducción subsecuente de la prevalencia de estos factores de riesgo cardiovascular en los grupos con mejor educación^28^ . Esto parece ser claro para obesidad, pero no es visible aún para hipertensión o diabetes mellitus tipo 2.

Nuestro estudio podría ayudar a definir estrategias de prevención dirigidas a la población más vulnerable de tener enfermedades crónicas, usualmente los más vulnerables económicamente; sin embargo, aún existe una mayor prevalencia de estos factores de riesgo en los de nivel socioeconómico medio-alto. La evidencia actual sugiere que el impacto de la transición se está viendo más en las zonas rurales que en las urbanas, especialmente en el caso de obesidad^29^ . Resultados previos sugieren que el Perú está en una etapa de postransición epidemiológica, en la cual más del 80% de las muertes son atribuidas a enfermedades no transmisibles^30^ , lo que puede significar un mayor reto al sistema de salud. Las intervenciones generadas deberían ser específicas de cada contexto, especialmente en aquellos sitios donde el sistema de salud puede no tener un acceso apropiado (zonas rurales y urbano marginales) que es donde el problema de las condiciones no transmisibles terminará alojándose.

Este trabajo usa información de un estudio de base poblacional con representatividad regional y nacional para evaluar las asociaciones de interés. Sin embargo, hay algunas limitaciones que deben ser resaltadas. Primero, al ser un estudio transversal, solo permitió determinar asociación y no causalidad entre exposiciones y efectos. Aunque la temporalidad podría ser un problema, vemos poco probable que exista causalidad reversa en este análisis. En segundo lugar, diabetes mellitus fue evaluado por autoreporte y no en forma objetiva. Se sabe que solo el 50% de la población con diabetes sabe que tiene el diagnóstico, y realizarse la prueba, probablemente, dependa del nivel socioeconómico de las personas, por lo que esto podría tener un efecto en las asociaciones reportadas. Sin embargo, nuestros hallazgos muestran resultados similares a otros estudios. Finalmente, otras variables de interés, potenciales confusores como la dieta o los niveles de actividad física, no fueron incluidos por no ser colectados en forma rutinaria por la Endes.

En conclusión, existe asociación diferencial entre el índice de bienestar, el nivel educativo y los marcadores de enfermedades no transmisibles: hay evidencia de asociación positiva entre el índice de bienestar y obesidad, hipertensión y diabetes mellitus tipo 2, mientras que el nivel educativo solo estuvo asociado de forma negativa a obesidad.
